# A single-center experience of haploidentical stem cell transplantation in hematological malignancies

**DOI:** 10.55730/1300-0144.5591

**Published:** 2022-11-22

**Authors:** Ümit Yavuz MALKAN, Hakan GÖKER, Haluk DEMİROĞLU, Fatma TEKİN, Buket AKDEMİR, Elifcan ALADAĞ KARAKULAK, Nilgün SAYINALP, İbrahim Celalettin HAZNEDAROĞLU, Osman İlhami ÖZCEBE, Yahya BÜYÜKAŞIK

**Affiliations:** Department of Hematology, Faculty of Medicine, Hacettepe University, Ankara, Turkey

**Keywords:** Haploidentical hematopoietic stem cell transplantation, Graft-versus-host disease, conditioning regimens

## Abstract

**Background/aim:**

Since well-designed prospective comparative trials are lacking, haploidentical hematopoietic stem cell transplantations approach should be based on the expertise of a particular center. In this study, we aimed to report the results and outcomes of patients who underwent haploidentical hematopoietic stem cell transplantation.

**Materials and methods:**

Thirty-nine patients who underwent transplantation in our clinic between 2015 and 2022 were retrospectively analyzed. Primary end point of this study is to find out the survival rates of the patients.

**Results:**

The overall survival of patients was 29.9 ± 4.9 months. The disease-free survival of the patients was 37.8 ± 5.7 months. The 3-year overall survival rate of the patients was %50 and the 3-year disease-free survival rate of the patients was %53. Nineteen patients were nonsurvivors among a total of 39 patients. Busulfan–fludarabine–thiotepa was the most frequently used conditioning regimen for transplantation. Busulfan–fludarabin–antithymocyte globulin regimen is the second preferred conditioning regimen. Cyclosporine–cyclophosphamide–mycophenolate mofetil was the most widely used graft-versus-host disease prophylaxis regimen. Sixteen patients had graft-versus-host disease, 28% of the patients had acute graft-versus-host disease, and 13% had chronic graft-versus-host disease. Gastrointestinal system consists of the most involved organs in graft-versus-host disease since 15% of the patients had gastrointestinal graft-versus-host disease. First-degree relatives (parent/child) were the most frequent donor source for haploidentical hematopoietic stem cell transplantation. Sepsis was the most frequent reason of death among transplant patients.

**Conclusion:**

In our center, we prefer to use high dose posttransplantation cyclophosphamide after haploidentical hematopoietic stem cell transplantation for graft-versus-host disease prophylaxis. With this approach, our center’s overall survival and disease-free survival rates are comparable and compatible with the literature findings.

## 1. Introduction

Allogeneic hematopoietic cell transplantation (HCT) is a potentially curative treatment for a wide variety of malignant and benign hematologic diseases. The pluripotent hematopoietic stem cell source is either the bone marrow or peripheral blood from a related or unrelated donor. Traditionally, the best results of allogeneic HCT have been achieved when the stem cell donor is a human leukocyte antigen (HLA)-matched sibling. However, the small family sizes and the 25% possibility that any sibling is fully HLA-matched to the patient, an HLA-matched sibling available for only about 30% of patients. HLA-matched or partially mismatched adult unrelated donors, umbilical cord blood stem cells, and partially HLA-mismatched, or HLA-haploidentical, related donors are alternative sources of donor grafts for patients who lack an HLA-matched sibling. Significant advances in haploidentical hematopoietic stem cell transplantations (HHSCT) have been achieved in the last decade and the use of haploidentical family donors is increasing [1, 2]. HLA-haploidentical stem cell transplantation with posttransplant cyclophosphamide has been commonly used worldwide [3]. This strategy was first developed in HCT with nonmyeloablative conditioning [4]. Due to the latest developments in HCT, donor type (HLA-haploidentical donor versus HLA-matched related or unrelated donor) may no longer be an important predictor of transplant outcome [3]. HHSCT could be used in malignant hematological disorders. GVHD is a major complication in allogeneic HCT [5]. Different conditioning regimens, stem cell sources, and graft-versus-host disease (GVHD) prophylaxis regimens have been proposed by different transplant authors [6]. Over the past several decades, numerous approaches to HHSCT have been developed. The substantial differences in study design and patients treated complicate any comparisons between these approaches. Since well-designed prospective comparative trials are lacking, HHSCT approach should be based on the expertise of a particular center. In this study, we aimed to report the results and outcomes of patients who underwent HHSCT.

## 2. Materials and methods

Thirty-nine patients who underwent HHSCT in our clinic between 2015 and 2022 were retrospectively analyzed. The inclusion criteria of the patients were to be at an age ≥ 18 years. The exclusion criteria of the study were as follows; age lower than 18 years and HHSCT procedure performed for hematologic benign disorders (such as aplastic anemia). HHSCT was performed to patients who were diagnosed with acute myeloid leukemia, acute lymphoblastic leukemia, chronic myeloid leukemia, myelodysplastic syndrome, myelofibrosis, Hodgkin lymphoma, multiple myeloma, and biphenotypic leukemia. Patients’ and donors’ sex, age, blood type, and cytomegalovirus (CMV) status, the disease type of patients, cytogenetic risk categories, disease last status, relapse or mortality, disease status before HHSCT and after 100th day, sixth month, and first year after HHSCT, conditioning regimens, amount of infused CD34+ cells and mononuclear cells, duration of neutrophil and platelet engraftments, Eastern Cooperative Oncology Group (ECOG) scores of the patients, GVHD characteristics and management, donor proximity to host, HLA mismatch rate, HHSCT complications, donor lymphocyte infusion, and amount of infused lymphocyte cells, BK virus infections and mortality reasons of the patients, were noted. Data of the patients were obtained from the hospital database. All of the ethical considerations were strictly followed in accordance with the 1964 Helsinki declaration. As a standard care/action of the hospitals of the Hacettepe University Hospitals, it has been recognized from the patient records that all of the studied patients had given informed consent at the time of hospitalization and before the administration of chemotherapy and other relevant diagnostic/therapeutic standard of care.

### 2.1. HHSCT conditioning regimens

Busulfan–fludarabine–thiotepa conditioning regimen consists of the following [7]; intravenous (iv) busulfan 3.2 mg/kg/day on days -4, -3, -2; fludarabine 40 mg/m^2^ on days -5, -4, -3, -2; thiotepa 5 mg/kg on days -7 and -6 followed by cyclophosphamide 50 mg/kg on days 3 and 4; mycophenolate mofetil 30 mg/kg/day; cyclosporine (CsA) 3 mg/kg/day (adjusted based on serum CsA levels); valacyclovir 3000 mg/day; metronidazole 1500 mg/day; fluconazole 400 mg/day.

Busulfan–fludarabine–antithymocyte globulin (ATG) conditioning regimen consists of the following [8]; Busulfan 3.2 mg/kg on days -5, -4, -3; fludarabin 50 mg/m^2^ on days -8, -7, -6, -5, -4, -3; ATG 8 mg/kg on days -2 and -1 followed by cyclophosphamide 50 mg/kg on days 3 and 4; mycophenolate mofetil 30 mg/kg/day; CsA 3 mg/kg/day (adjusted based on serum CsA levels); valacyclovir 3000 mg/day; metronidazole 1500 mg/day; fluconazole 400 mg/day.

### 2.2. Statistical analysis

Statistical analyses were executed with the SPSS software v.25. At first, categorical and continuous variables were defined. The descriptive statistics of the categorical variables were given in the tables. The continuous variables were presented as median (minimum–maximum). Primary end point of this study is to find out the overall survival (OS) and (DFS) rates of the patients. OS was calculated from diagnosis to the date of death due to any cause. DFS was analyzed in complete remission (CR) patients from the date of CR attainment to relapse or death in remission. Patients who are survivors and those who did not relapse or nonsurvivors during the first CR were censored at the last follow-up for OS and DFS computations, respectively. OS and DFS are calculated with the Kaplan–Meier method.

### 2.3. Ethical board approval

This study was approved by Hacettepe University Ethical Board on 31.05.2022 with the approval number of GO 22/544.

## 3. Results

**Figure 1:**
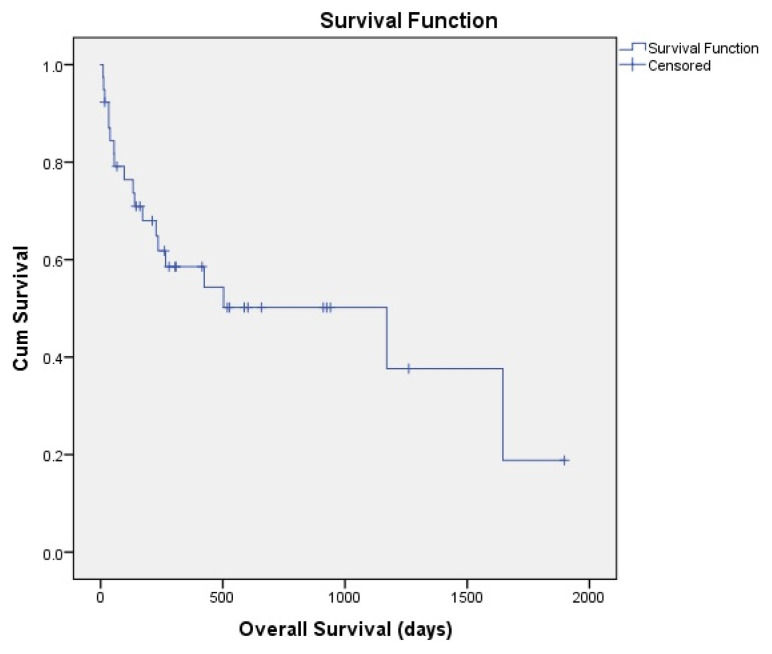
Overall survival of the patients.

**Figure 2:**
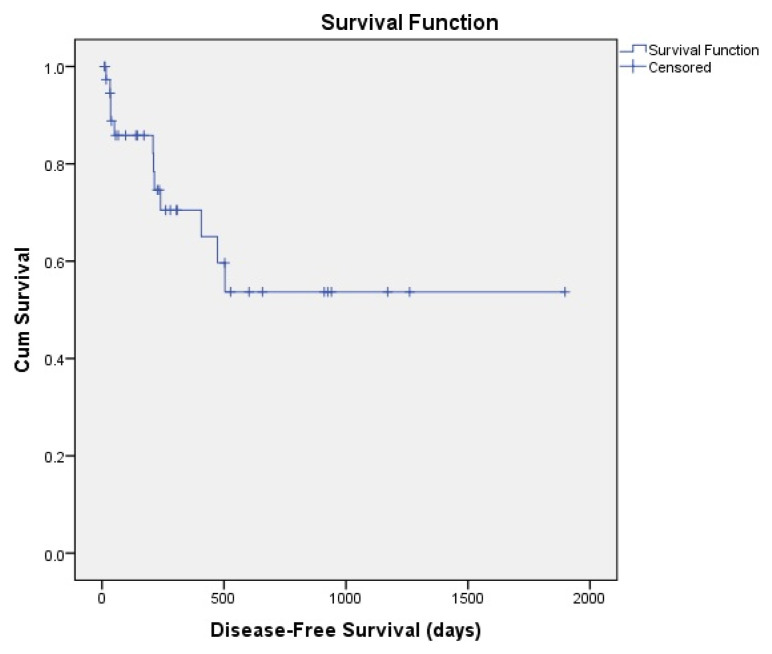
Disease-free survival of the patients.

A total of 39 patients were analyzed. The main parameters of the patients were given in Table 1. The median age of the patients was 45 years, whereas the median age for donors was 32. Male patients were more than female patients; similarly, male donors were more than female donors. The transplant-related parameters of the patients are given in Table 2. The overall survival of patients was 897 ± 147 days (Figure 1). The disease-free survival of the patients was 1135 ± 171 days (Figure 2). The 3-year OS rate of the patients was %50 and the 3-year DFS rate of the patients was %53. Nineteen patients were nonsurvivors among a total of 39 patients. Most of the patients who underwent HHSCT were acute myeloid leukemia patients. Acute lymphoblastic leukemia was the second frequent disease type among the HHSCT patients. HLA compliance rate between the patients and donor was in the range of 5/10–8/10. Majority of the patients had intermedia cytogenetic risk category. The median duration for neutrophil engraftment was 15 days and the median duration for platelet engraftment was 17 days. The median amount of infused CD34+ cells was 11.4 × 10^6^/kg and the median number of infused mononuclear cells was 6.6 × 10^8^/kg. Twelve patients had relapsed after HHSCT. On the 100th day after HHSCT, most of the patients (n:24) had complete disease remission. At 6th month evaluation, 19 patients had sustained CR. At the first year, only 12 patients had remained in CR. Sixteen patients had GVHD, 28% of all patients had acute GVHD (n:11) and 13% of all patients had chronic GVHD (n:5). Gastrointestinal system is the most involved organ in GVHD since 15% of patients had gastrointestinal GVHD (n:6). Liver and mouth GVHD were seen in four patients each. Nine patients (23%) had grade 3 severe GVHD. Steroid plus cyclosporine is the most frequently selected treatment agent for GVHD treatment (%35.7). Six patients responded to the GVHD treatment agents whereas in 10 patients no response was observed with GVHD treatment. Twenty-three patients were in disease remission before HHSCT. Busulfan–fludarabine–thiotepa was the most frequently used conditioning regimen for HHSCT. Busulfan–Fludarabin–ATG regimen is the second preferred conditioning regimen. In all of the patients, we have administrated posttransplant high dose cyclophosphamide in order to overcome GVHD. Cyclosporine + cyclophosphamide + mycophenolate mofetil was the most widely used GVHD prophylaxis regimen. First-degree relatives (parent/child) were the most frequent donor source for HHSCT. O Rh+ blood type was the most frequent among HHSCT patients whereas A Rh+ was the most frequent in donors. Only in one patient CMV IgG was negative. On the other hand, two donors were CMV IgG negative. Veno-occlusive disease, mucositis, neutropenic fever and diarrhea was the most frequently encountered complication after HHSCT. Pulmonary thromboembolism (n:1), dyspnea (n:3), CMV infection (n:2), hypotension (n:1), urinary tract infection (n:1), rash, veno-occlusive disease (n:3), neutropenic fever (n:4), mucositis (n:3), and acute renal injury (n:1) were the other complications in our HHSCT patients. Donor lymphocyte infusion was performed in 4 patients. BK virus was detected in 4 HHSCT patients. Sepsis was the most frequent reason of death with %63 (n:12) among the nonsurvivor HHSCT patients.

## 4. Discussion

Near universal availability of highly motivated donors, adequate doses of hematopoietic stem cells, availability of the donor for repetitive donations of hematopoietic stem cells or lymphocytes to treat relapse and graft-versus-leukemia effect are among the advantages of HHSCT. Patients have approximately 2.7 potential HLA-haploidentical donors from first-degree relatives [9]. Likely, in our center, first-degree relatives (parent/child) were the most frequent donor source for HHSCT. HLA-haploidentical grafts have enough doses of hematopoietic stem cells for transplantation and of memory T cells for immune reconstitution. Similarly, we did not encounter engraftment failure in our HHSCT patients. In 4 of our HHSCT patients, we have performed donor lymphocyte infusion, which is an advantage of HHSCT. For patients with high-risk acute leukemia, HHSCT could be related with a stronger graft-versus-leukemia effect compared with HLA-matched sibling HCT, resulting in a lower cumulative incidence of relapse [10] and an improved overall survival [11].

On the other hand, HHSCT may also have risk that should be carefully managed. According to the International Bone Marrow Transplant Registry, when compared with HLA-matched sibling HCT, two HLA antigen-mismatched related donor transplants resulted in higher rates of the following adverse transplant outcomes which are transplant-related mortality (55% versus 21% at three years among patients with leukemia), graft failure (16% versus 1%), grade II to IV acute GVHD (56% versus 29%), severe (grade III/IV) acute GVHD (36% versus 13%) and chronic GVHD (60% versus 42%) [12]. In order to overcome these problems, attempts at T cell depletion of the donor graft reduced the incidence of acute GVHD, but at the cost of increased incidence of graft rejection, and did not improve leukemia-free survival [13].

There are several strategies for HHSCT procedure. In the Asian countries, the GIAC approach is performed. GIAC has four main components which are GCSF-stimulation of the donor; intensified immunosuppression through posttransplantation cyclosporine (CsA), mycophenolate mofetil (MMF), and short-course methotrexate. Moreover, ATG is added to conditioning to avoid GVHD and stimulate engraftment. The GIAC protocol may achieve complete engraftment, acceptable nonrelapse mortality with favorable disease-free survival [14, 15]. On the other hand, high rates of severe acute and chronic GVHD are related with GIAC approach [16].

High-dose posttransplantation cyclophosphamide (PTCy) is a strategy for HHSCT that is relatively inexpensive and requires no graft manipulation. With this regimen, retrospective studies proposed that the significant HLA disparity of HHSCT is not related with increased acute GVHD or worsened progression-free survival (PFS) in acute leukemia or lymphomas [17, 18]. The feasibility and efficacy of PTCy in HHSCT procedure are among the benefits of this strategy. In our clinic, PTCy strategy is used for GVHD prophylaxis and preferred for HHSCT procedures. On the other hand, there may be some drawbacks of this strategy. In previous analysis, posttransplant cyclophosphamide is associated with increased cytomegalovirus infection [19]. However, despite the usage of PTCy in our HHSCT patient, CMV infection detected only in 2 patients among a total of 39 in our study. Therefore, it may be suggested that the high of CMV infection in PTCy strategy can be managed with appropriate CMV infection prophylaxis.

Busulfan plus cyclophosphamide (BuCy) is the conventional conditioning regimen for HCT for young, fit patients with AML. The thiotepa–busulfan–fludarabine (TBF) protocol has recently resulted with promising outcome in cord blood and HHSCT. In a recent comparison, TBF was found to represent a valid myeloablative conditioning regimen providing significantly lower relapse and similar survival when compared with BuCy [20]. Patients in first remission seem to benefit the most from this protocol, as in this subgroup an affinity for better leukemia-free-survival was detected when compared with BuCy [20]. In the present study, the most widely used conditioning regimen was TBF protocol.

Severe infections and their attributable mortality are major complications in recipients of allogeneic HCT. In a previous study bacterial infections were found to be the most common causes of infection-related mortality (51%) [21]. Severe infections are the most common causes of nonrelapse mortality after HHSCT with PTCy, with a reemergence of gram-negative bacilli as the most lethal pathogens [21]. In another recent study aiming to investigate the rates of infection-related mortality and other complications following haploidentical vs nonhaploidentical transplant, despite the use of identical antimicrobial prophylactic and treatment agents, haploidentical recipients were found to have considerably increased rates of 100-day and 1-year infection-related mortality as well as numerous other infectious complications [22]. The incidence of community respiratory viral infections was found to be higher for patients receiving PTCy, regardless of donor type in HHSCT [23]. Moreover, an increased incidence of bacterial, fungal, or viral infections is found in HHSCT compared to related, unrelated, or cord blood transplantations. Neutropenia and use of systemic steroid for GVHD and delayed immune reconstitution are important risk factors for infection after haploidentical HSCT [24]. Similarly, in the present study, the most known cause of mortality among HHSCT patients is sepsis.

PTCy combined with calcineurin inhibitors, such as cyclosporine A (CsA) or tacrolimus, is a well-established GVHD prophylaxis in the setting of HHSCT [25]. In terms of GVHD prophylaxis patients with hematological malignancies undergoing haploidentical stem cell transplantation with PTCy and mycophenolate mofetil (MMF), combined with cyclosporine A has shown to be a sufficient strategy in terms of engraftment, GVHD incidence, and survival [26]. The most preferred regimen for GVHD prophylaxis used is CsA–cyclophosphamide–MMF which is similar with the literature.

Worldwide Network for Blood and Marrow Transplantation stated that there are three major study groups and experiences worldwide which are Asian, European, and North American experiences [27]. PTCy was first developed by Schwartz and Dameshek [28]. They found that an immunogenic antigen exposure stimulates the increase of antigen-specific B cells and T cells, timely use of the cytotoxic drug will selectively inhibit the antigen-responsive lymphocytes while sparing lymphocytes specific for other antigens. Berenbaum showed that cyclophosphamide may increase the survival of rat skin allografts if the drug was given approximately one to three days after graft placement [29]. With the regimen that is proposed and used at Johns Hopkins in Baltimore including Cy 50 mg/kg/day on days 3 and 4 followed by G-CSF 5 μg/kg/day, the incidences of acute and chronic GVHD were very low, and nonrelapse mortality was found as 17% in the long term. Overall and event-free survival rates at 5 years after HHSCT were in the approximately 40% and 30%, respectively. The outcomes of reduced-intensity conditioning and HHSCT with PTCy are nearly equivalent to the outcomes of patients receiving grafts from HLA-matched donors [30]. High-dose PTCy for GVHD prophylaxis has now been widely used with favorable outcomes after myeloablative and nonmyeloablative conditioning [31, 32].

Our study has a few limitations. Firstly, the retrospective design is the major limitation of the study. Secondly, relatively small number of study participants is another limiting factor. On the other hand, the most important finding of the current study is the real-life favorable outcome of HHSCT with PTCy strategy, busulfan–fludarabine–thiotepa and busulfan–fludarabine–antithymocyte globulin conditioning regimens.

## 5. Conclusion

The main advantages of HHSCT are the availability of highly motivated donors, rapid availability and relatively low cost of the stem cell source, adequate doses of hematopoietic stem cells for HHSCT and immune reconstitution, and the availability of the donor for repeated donations of hematopoietic stem cells or lymphocytes to treat relapse. The main problem of HHSCT is in the absence of appropriate prophylactic procedures, high incidences of fatal graft rejection or severe or fatal GVHD. In our center, we prefer to use high-dose PTCy after HHSCT for GVHD prophylaxis. With this approach, our center’s overall survival and disease-free survival rates are comparable and compatible with the literature findings. HHSCT has more infection risk compared to related, unrelated, or cord blood transplantations. Likely, sepsis and infections are the most frequent causes of death in our HHSCT patients. In the context of long-term immune recovery and impaired immunity due to use systemic steroid for GvHD in HHSCT, preventive and treatment strategies are needed to improve long-term outcomes in HHSCT patients. HHSCT with PTCy overcome the HLA-barrier in transplantation, but infection prophylaxis is needed to avoid mortality. Future prospective larger controlled clinical studies are to delineate the definitive role of HHSCT in this new transplant era.

### Informed consent

This study is approved by Hacettepe University Ethical Board on 31.05.2022 with the approval number of GO 22/544. All of the studied patients had given informed consents at the time of hospitalization

## Figures and Tables

**Table 1:** The main parameters of the patients.

Parameters	Value
Patient sex (female/male)	16/23
Patient age (years)	45 (18–68)
Donor sex (female/male)	9/30
Donor age (years)	32 (20–65)
Type of disease (AML/ALL/MDS/KML/PMF/HL/MM/BAL)	26/6/2/1/1/1/1/1
Cytogenetic risk category (Favorable/Moderate/Unfavorable/NR)	4/23/1/11
Status before transplantation (CR/active disease)	23/16
Mortality (Yes/No)	19/20
Last disease status (CR/Relapse/Active Disease/Exitus)	16/3/1/19
Chemotherapy for relapse (EMA/Vidaza-Venetoclax/Blinatumomab/Inotuzumab/Flag-IDA/Dara-VD/Venetoclax/NA-NR)	1/2/1/1/1/1/1/3
ECOG score (0–1/2–3–4)	38/1
Patient CMV IgG (positive /negative)	38/1
Donor CMV IgG (positive /negative)	37/2

**Abbreviations**: AML: acute myeloid leukemia, ALL: acute lymphoblastic leukemia, MDS: myelodysplastic syndrome, KML: chronic myeloid leukemia, PMF: primary myelofibrosis, HL: hodgkin lymphoma, MM: multiple myeloma, BAL: Biphenotypic acute leukemia, CMV: cytomegalovirus, EMA: Etoposide-Mitoxantrone-Cytarabine, Flag-IDA: Fludarabine-Idarubisin- Cytarabine, ECOG: Eastern Cooperative Oncology Group, CR: complete response, PR: partial response

**Table 2:** Parameters of the patients related with transplantation.

Parameters	Value
Relapse after transplant(Yes/No)	12/27
100-day disease status (CR/PR/Ex/Relapse/Active Disease/NR)	24/1/6/1/1/6
6-month disease status (CR/NR-NA)	19/20
1-year disease status (CR/Ex/Relapse/NR-NA)	12/2/1/24
Conditioning regimens (Bu-Flu-ATG/Bu-Flu-Thiotepa/others)	10/27/2
Infused CD34 cells × 10^6^	11.4 (4.7–27.6)
Infused mononuclear cell × 10^8^	6.6 (2.2–13.0)
Neutrophil engraftment duration (days)	15 (10–18)
Platelet engraftment duration (days)	17 (9–44)
GVHD (Yes/No/NR)	16/8/15
GVHD Type (Acute/Chronic/Acute onset chronic)	7/5/4
Skin GVHD (Yes/No)	11/5
Gastrointestinal GVHD (Yes/No)	6/10
Liver GVHD (Yes/No)	4/12
Eye GVHD (Yes/No)	2/14
Mouth GVHD (Yes/No)	4/12
Pulmonary GVHD (Yes/No)	1/15
GVHD degree (1/2/3)	5/2/9
GVHD prophylaxis regimens (CsA-CP-MMF/ CsA-CP-ATG/ CsA-MTX-ATG/ Tacrolimus-MTX)	36/1/1/1
GVHD treatment regimens (steroid/CsA/steroid+CsA/tacrolimus/steroid+tacrolimus/ steroid+tacrolimus+MMF+Somatostatin/eye drops)	3/1/5/1/2/1/1
Acute GVHD treatment response (Complete / partial/ none)	3/3/10
Donor proximity (Sibling/parent or child/ cousin or nephew)	17/19/3
Patient blood type (0 Rh+/ A Rh+/ B Rh+/ AB Rh+/A Rh-/B Rh-)	17/14/2/2/3/1
Donor blood type (0 Rh+/ A Rh+/ B Rh+/ AB Rh+/0 Rh-/A Rh-/B Rh-)	13/18/1/1/3/2/1
Complications (VOD/Mucositis/NPA/PTE/dyspnea/diarrhea/CMV/hypotension/urinary tract infection/rash/acute renal injury)	3/3/4/1/1/3/2/1/1/1/1
Donor lymphocyte infusion (Yes/No)	4/35
Infused lymphocyte cells × 10^7^	4.6 (3.8–7.5)
BK virus infection (Yes/No)	4/35
Mortality reason (Sepsis/Relapse/GVHD)	12/4/3

**Abbreviations**: NR: not reached, NA: not applicable, Bu: Busulfan, Flu: Fludarabine, ATG: Anti-thymocyte globulin, CsA: cyclosporine, CP: Cyclophosphamide, MTX: Methotrexate, MMF: Mycophenolate mofetil, CR: complete response, VOD: veno-occlusive disease, NPA: neutropenic fever, GVHD: graft-versus-host-disease

## References

[1] Kanakry CG Fuchs EJ Luznik L Modern approaches to HLA-haploidentical blood or marrow transplantation Nature Reviews Clinical Oncology 2016 13 10 24 10.1038/nrclinonc.2015.128 PMC4695979 26305035

[2] Passweg JR Baldomero H Bader P Bonini C Cesaro S Hematopoietic stem cell transplantation in Europe 2014: more than 40 000 transplants annually Bone Marrow Transplantation 2016 51 786 792 10.1038/bmt.2016.20 26901709 PMC4895175

[3] Sugita J HLA-haploidentical stem cell transplantation using posttransplant cyclophosphamide International journal of hematology 2019 110 30 38 10.1007/s12185-019-02660-8 31104211

[4] O’Donnell PV Luznik L Jones RJ Vogelsang GB Leffell MS Nonmyeloablative bone marrow transplantation from partially HLA-mismatched related donors using posttransplantation cyclophosphamide Biology of Blood and Marrow Transplantation 2002 8 377 386 10.1053/bbmt.2002.v8.pm12171484 12171484

[5] Aladağ Karakulak E Demİroğlu H Malkan UY Akman U Göker H Assessment of ST2 and Reg3a levels in patients with acute graft-versus-host disease after allogeneic hematopoietic stem cell transplantation Turkish Journal of Medical Sciences 2021 51 355 358 10.3906/sag-2007-17 32927932 PMC7991887

[6] Sengsayadeth S Savani BN Blaise D Mohty M Haploidentical transplantation: selecting optimal conditioning regimen and stem cell source Seminars in Hematology 2016 53 111 114 10.1053/j.seminhematol.2016.01.012 27000735

[7] Pagliardini T Castagna L Harbi S Porta MD Rey J Thiotepa, Fludarabine, and Busulfan Conditioning Regimen before T Cell-Replete Haploidentical Transplantation with Post-Transplant Cyclophosphamide for Acute Myeloid Leukemia: A Bicentric Experience of 100 Patients Biology of Blood and Marrow Transplantation 2019 25 1803 1809 10.1016/j.bbmt.2019.05.014 31128325

[8] El-Cheikh J Devillier R Dulery R Massoud R Al Chami F Impact of Adding Antithymocyte Globulin to Posttransplantation Cyclophosphamide in Haploidentical Stem-Cell Transplantation Clinical Lymphoma, Myeloma & Leukemia 2020 20 617 623 10.1016/j.clml.2020.04.003 32457025

[9] Gragert L Eapen M Williams E Freeman J Spellman S HLA match likelihoods for hematopoietic stem-cell grafts in the U.S. registry New England Journal of Medicine 2014 371 339 348 10.1056/NEJMsa1311707 25054717 PMC5965695

[10] Kanda Y Chiba S Hirai H Sakamaki H Iseki T Allogeneic hematopoietic stem cell transplantation from family members other than HLA-identical siblings over the last decade (1991–2000) Blood 2003 102 1541 1547 10.1182/blood-2003-02-0430 12714500

[11] Wang Y Liu DH Xu LP Liu KY Chen H Superior graft-versus-leukemia effect associated with transplantation of haploidentical compared with HLA-identical sibling donor grafts for high-risk acute leukemia: an historic comparison Biology of Blood and Marrow Transplantation 2011 17 821 830 10.1016/j.bbmt.2010.08.023 20831895

[12] Szydlo R Goldman JM Klein JP Gale RP Ash RC Results of allogeneic bone marrow transplants for leukemia using donors other than HLA-identical siblings Journal of Clinical Oncology 1997 15 1767 1777 10.1200/JCO.1997.15.5.1767 9164184

[13] Ash RC Horowitz MM Gale RP van Bekkum DW Casper JT Bone marrow transplantation from related donors other than HLA-identical siblings: effect of T cell depletion Bone Marrow Transplantation 1991 7 443 452 1873591

[14] Huang XJ Liu DH Liu KY Xu LP Chen H Haploidentical hematopoietic stem cell transplantation without in vitro T-cell depletion for the treatment of hematological malignancies Bone Marrow Transplantation 2006 38 291 297 10.1038/sj.bmt.1705445 16883312

[15] Liu D Huang X Liu K Xu L Chen H Haploidentical hematopoietic stem cell transplantation without in vitro T cell depletion for treatment of hematological malignancies in children Biology of Blood and Marrow Transplantation 2008 14 469 477 10.1016/j.bbmt.2008.02.007 18342790

[16] Wang Y Liu QF Xu LP Liu KY Zhang XH Haploidentical vs identical-sibling transplant for AML in remission: a multicenter, prospective study Blood 2015 125 3956 3962 10.1182/blood-2015-02-627786 25940714

[17] Bashey A Zhang X Sizemore CA Manion K Brown S T-cell-replete HLA-haploidentical hematopoietic transplantation for hematologic malignancies using post-transplantation cyclophosphamide results in outcomes equivalent to those of contemporaneous HLA-matched related and unrelated donor transplantation Journal of Clinical Oncology 2013 31 1310 1316 10.1200/JCO.2012.44.3523 23423745

[18] Kanate AS Mussetti A Kharfan-Dabaja MA Ahn KW DiGilio A Reduced-intensity transplantation for lymphomas using haploidentical related donors vs HLA-matched unrelated donors Blood 2016 127 938 947 10.1182/blood-2015-09-671834 26670632 PMC4760094

[19] Goldsmith SR Abid MB Auletta JJ Bashey A Beitinjaneh A Posttransplant cyclophosphamide is associated with increased cytomegalovirus infection: a CIBMTR analysis Blood 2021 137 3291 3305 10.1182/blood.2020009362 33657221 PMC8351903

[20] Saraceni F Beohou E Labopin M Arcese W Bonifazi F Thiotepa, busulfan and fludarabine compared to busulfan and cyclophosphamide as conditioning regimen for allogeneic stem cell transplant from matched siblings and unrelated donors for acute myeloid leukemia American Journal of Hematology 2018 93 1211 1219 10.1002/ajh.25225 30033639

[21] Esquirol A Pascual MJ Kwon M Pérez A Parody R Severe infections and infection-related mortality in a large series of haploidentical hematopoietic stem cell transplantation with post-transplant cyclophosphamide Bone Marrow Transplantation 2021 56 2432 2444 10.1038/s41409-021-01328-4 34059802 PMC8165955

[22] Chang J Hsiao M Blodget E Akhtari M Increased risk of 100-day and 1-year infection-related mortality and complications in haploidentical stem cell transplantation Journal of Blood Medicine 2019 10 135 143 10.2147/JBM.S201073 31191064 PMC6526927

[23] Mulroney CM Abid MB Bashey A Chemaly RF Ciurea SO Incidence and impact of community respiratory viral infections in post-transplant cyclophosphamide-based graft-versus-host disease prophylaxis and haploidentical stem cell transplantation British Journal of Haematology 2021 194 145 157 10.1111/bjh.17563 34124796 PMC8853845

[24] Atilla E Atilla PA Bozdağ SC Demirer T A review of infectious complications after haploidentical hematopoietic stem cell transplantations Infection 2017 45 403 411 10.1007/s15010-017-1016-1 28417421

[25] Robinson TM O’Donnell PV Fuchs EJ Luznik L Haploidentical bone marrow and stem cell transplantation: experience with post-transplantation cyclophosphamide Seminars in Hematology 2016 53 90 97 10.1053/j.seminhematol.2016.01.005 27000732 PMC4806368

[26] Hernani R Piñana JL Pérez A Quintero A Montoro J Sirolimus versus cyclosporine in haploidentical stem cell transplantation with posttransplant cyclophosphamide and mycophenolate mofetil as graft-versus-host disease prophylaxis eJHaem 2021 2 236 248 10.1002/jha2.183 35845283 PMC9175741

[27] Apperley J Niederwieser D Huang XJ Nagler A Fuchs E Haploidentical Hematopoietic Stem Cell Transplantation: A Global Overview Comparing Asia, the European Union, and the United States Biology of Blood and Marrow Transplantation 2016 22 23 26 10.1016/j.bbmt.2015.11.001 26551633

[28] Schwartz R Dameshek W Drug-induced immunological tolerance Nature 1959 183 1682 1683 10.1038/1831682a0 13666859

[29] Berenbaum M Brown I Prolongation of homograft survival in mice with single doses of cyclophosphamide Nature 1963 200 84 10.1038/200084a0 14074645

[30] Armand P Gibson CJ Cutler C Ho VT Koreth J A disease risk index for patients undergoing allogeneic stem cell transplantation Blood 2012 120 905 913 10.1182/blood-2012-03-418202 22709687 PMC3412351

[31] Solomon SR Sizemore CA Sanacore M Zhang X Brown S Haploidentical transplantation using T cell replete peripheral blood stem cells and myeloablative conditioning in patients with high-risk hematologic malignancies who lack conventional donors is well tolerated and produces excellent relapse-free survival: results of a prospective phase II trial Biology of Blood and Marrow Transplantation 2012 18 1859 1866 10.1016/j.bbmt.2012.06.019 22863841

[32] Raiola AM Dominietto A di Grazia C Lamparelli T Gualandi F Unmanipulated haploidentical transplants compared with other alternative donors and matched sibling grafts Biology of Blood and Marrow Transplantation 2014 20 1573 1579 10.1016/j.bbmt.2014.05.029 24910379

